# Alterations in serum amino-acid profile in the progression of colorectal cancer: associations with systemic inflammation, tumour stage and patient survival

**DOI:** 10.1038/s41416-018-0357-6

**Published:** 2018-12-19

**Authors:** Päivi Sirniö, Juha P. Väyrynen, Kai Klintrup, Jyrki Mäkelä, Toni Karhu, Karl-Heinz Herzig, Ilkka Minkkinen, Markus J. Mäkinen, Tuomo J. Karttunen, Anne Tuomisto

**Affiliations:** 1 Cancer and Translational Medicine Research Unit, University of Oulu, POB 5000, 90014 Oulu, Finland; 2 Oulu University Hospital and Medical Research Center Oulu, POB 21, 90029 Oulu, Finland; 3 Research Unit of Surgery, Anesthesia and Intensive Care, University of Oulu, POB 5000, 90014 Oulu, Finland; 4 Department of Physiology, Research Unit of Biomedicine and Biocenter Oulu, University of Oulu, POB 5000, 90014 Oulu, Finland; 5 Department of Gastroenterology and Metabolism, Poznan University of Medical Sciences, ul. Szpitalna 27/33, 60-572 Poznan, Poland

**Keywords:** Colorectal cancer, Cancer metabolism

## Abstract

**Background:**

Cancer cachexia is a complex wasting syndrome affecting patients with advanced cancer, with systemic inflammation as a key component in pathogenesis. Protein degradation and release of amino acids (AAs) in skeletal muscle are stimulated in cachexia. Here, we define factors contributing to serum AA levels in colorectal cancer (CRC).

**Methods:**

Serum levels of nine AAs were characterised in 336 CRC patients and their relationships with 20 markers of systemic inflammatory reaction, clinicopathological features of cancers and patient survival were analysed.

**Results:**

Low serum glutamine and histidine levels and high phenylalanine levels associated with indicators of systemic inflammation, including high modified Glasgow Prognostic Score, high blood neutrophil/lymphocyte ratio and high serum levels of CRP, IL-6 and IL-8. Low levels of serum glutamine, histidine, alanine and high glycine levels also associated with advanced cancer stage and with poor cancer-specific survival in univariate analysis.

**Conclusions:**

In CRC, serum AA levels are associated with systemic inflammation and disease stage. These findings may reflect muscle catabolism induced by systemic inflammation in CRC.

## Introduction

Colorectal cancer (CRC) is the third most common cancer worldwide and a major cause of mortality.^1^ It is estimated that approximately half of CRC patients develop cachexia, a complex wasting syndrome characterised by progressive loss of skeletal muscle and adipose tissue.^2^ The international consensus definition of cachexia is an ongoing loss of skeletal muscle mass—with or without loss of fat mass—that cannot be fully reversed by conventional nutritional support and that leads to progressive functional impairment.^3^ Cachexia is associated with involuntary weight loss, systemic inflammation, anorexia, insulin resistance and fatigue.^3^ It not only has a significant impact on quality of life but also is associated with poor response to treatment, increased toxicity from chemotherapy and decreased survival.^3^ Importantly, targeting host wasting can prolong survival, irrespective of tumour progression.^4^ Thus, therapies preventing or delaying cachexia in parallel with the specific oncological regimens could improve patient outcome rendering the understanding of the mechanisms of cancer cachexia of vast importance.

Mechanisms underlying cancer-associated cachexia are inadequately understood, but cachexia is associated with persistent elevations in liver-secreted acute phase response proteins, including serum CRP.^5,6^ In addition, several pro-inflammatory cytokines such as interleukin-6 (IL-6) are known to promote cachexia,^7^ and IL-6-blocking antibody can attenuate cachexia progression in murine model.^8^ We have shown that the levels of several cytokines, including IL-6, are increased in CRC patients,^9^ especially in patients with systemic inflammation as determined by the modified Glasgow Prognostic Score (mGPS),^9^ a measure based on the circulating levels of CRP and albumin.^10^ Thus, it has been hypothesised that mGPS could serve as an indirect measurable cachexia marker in CRC patients.^10^

Muscle wasting, characteristic feature of cachexia, is preceded by metabolic alterations and, hence, serum metabolites, including amino acids (AAs), are potential markers for the early detection of cancer cachexia. Indeed, altered circulating concentrations of AAs have been found in many diseases.^11,12^ Protein degradation and release of AAs are stimulated in skeletal muscle on demand. AAs can be utilised as a substrate in various pathways to produce proteins, small peptides, fatty acids and glucose, and as precursors for other compounds, for example, purines and pyrimidines. Glutamine is the most abundant AA in the body, and in the serum, glutamine, mainly released from skeletal muscle, functions as an important inter-organ carbon and nitrogen transporter.^13^ In critically ill patients, protein degradation and muscle loss are increased, glutamine consumption is increased and thus circulating glutamine level is decreased.^12^ A few studies have identified disease-associated alterations in the amino-acid profile as a diagnostic biomarker for CRC.^14–16^ However, a more detailed analysis relative to clinicopathological features, systemic inflammatory markers and survival in CRC could improve our knowledge on CRC progression. Therefore, in this study, we measured serum levels of nine AAs in 336 CRC patients and analysed their relationships with clinicopathological features, 20 systemic inflammatory markers, and survival.

## Materials and methods

### Patients

The study was based on 357 newly diagnosed CRC patients operated in the Oulu University Hospital between 2006 and 2014, who had signed an informed consent to participate and were eligible to the study.^9,17^ The patients with earlier or simultaneously diagnosed other malignant diseases were excluded. Preoperative blood samples were collected from 341 patients. Five (1.5%) cases were excluded owing to insufficient sample material. Clinical data were collected from the clinical records and a questionnaire. The follow-up data were acquired from the clinical records and Statistics Finland.^18,19^ Study endpoints were disease-free survival, cancer-specific survival (CSS) and overall survival (OS).^17^ The study was accepted by the Ethical Committee of the Oulu University Hospital (58/2005, 184/2009) and performed according to the principles of the Declaration of Helsinki. The REMARK guidelines were taken into account.^20^

### Histopathological analysis

The tumours were staged according to TNM8 classification and graded according to the WHO2010 criteria.^21^

### Immunohistochemistry

Tissue microarrays were utilised in the immunohistochemical analyses. The arrays included 1–4 cores of 3.0 mm diameter, depending on the size of the tumour, from the invasive margin (IM) and the tumour centre (CT).^23^ Mismatch repair (MMR) enzyme screening status for MLH1, MSH2, MSH6 and PMS2 was evaluated with immunohistochemistry, as described earlier.^17,24,25^ BRAF V600E specific VE1 immunohistochemistry was performed.^17,26^ To determine the properties of local inflammatory reaction, immunohistochemistry for five inflammatory cell markers (CD3, T cells; CD8, cytotoxic T cells; FoxP3, regulatory T cells; mast cell tryptase, mast cells; and neutrophil elastase, neutrophilic granulocytes) was conducted.^27,28^ For immune cell counting, images were captured from the CT and the IM and the cell densities were counted using a computer assisted cell counting method.^29^ The fraction of proliferating cancer cells (Ki-67 score) was assessed with Ki-67 immunohistochemistry.^22^

### Analysis of serum samples

Nuclear magnetic resonance metabolomics platform, equipped with Bruker AVANCE III 500 MHz and Bruker AVANCE III 600 MHz spectrometers (Bruker, Billerica, MA, USA), was used to analyse serum alanine, glutamine, glycine, histidine, isoleucine, leucine, valine, phenylalanine and tyrosine levels.^30^ Of 336 CRC serum samples analysed, automatic sample and measurement quality control accepted 326 (glycine), 333 (glutamine), 335 (alanine, histidine, isoleucine, leucine, valine and tyrosine) and 336 (phenylalanine) measurements. Blood leucocyte, neutrophil, monocyte and lymphocyte counts, serum CRP levels and serum albumin levels were measured.^9,17,24^ Serum concentrations of 13 cytokines were measured from patients operated between April 2006 and January 2010 (*n* = 144).^9^

### Statistical analyses

The statistical analyses were conducted using IBM SPSS Statistics for Windows version 22.0 (IBM Corporation, Armonk, NY, USA). Normally distributed continuous variables are presented as mean (standard deviation, SD). Statistical significances of the differences in serum alanine, glutamine, glycine, histidine, isoleucine, leucine, valine, phenylalanine and tyrosine levels between the different categorical variables were analysed by independent samples *t* test or one-way analysis of variance. Correlations between two continuous variables were presented as Pearson correlation coefficients (*r*). The two-dimension (2D) visualisation was created with Cytoscape, an open source software platform for network analysis, utilising the Prefuse force-directed algorithm weighted by the statistical significances of the correlations between individual variables.^31^ To decrease the number of edges and make the figure more clear, only edges with *p* < 0.01 are shown. Multiple linear regression analysis of the correlation of serum AA levels with selected clinicopathological factors was conducted. Correlation matrix plot was drawn with R version 3.5.0 (R Foundation for Statistical Computing, Vienna, Austria) using the package corrplot. Receiver operating characteristics (ROC) analysis was used to determine optimal cut-off values in detecting patients, who survived in 120-month follow-up (CSS). Log-rank test was utilised in the univariate survival analyses. Cox’s proportional hazards regression model was used to assess the independent prognostic significance of AAs. Considering multiple hypothesis testing, a two-tailed *p* < 0.01 was considered statistically significant.

## Results

### General characteristics

The characteristics of patients are shown in Table 1. Of the analysed AAs in our CRC cohort, the levels of glutamine were highest, followed by alanine, glycine and valine (Table 1). The AA levels, especially isoleucine, leucine and valine, had high intercorrelation (Figure S1). The international plasma AA reference values and how AA levels in CRC patients fit in these ranges are described in table S1.^32^ CRC patients were most often deficient of glutamine and branched AAs leucine, isoleucine and valine. Moreover, phenylalanine levels were above the range in 96 CRC patients.

**Table 1:** Characteristics of colorectal cancer patients

Colorectal cancer patients (*n* = 336)	
Age, mean (SD)	68.2 (11.5)
Gender	
Male	180 (53.6%)
Female	156 (46.4%)
Preoperative radiotherapy or chemoradiotherapy	
No	267 (79.5%)
Yes	69 (20.5%)
Tumour location	
Proximal colon	117 (34.8%)
Distal colon	71 (51.1%)
Rectum	148 (44.0%)
WHO grade	
Grade 1	73 (21.9%)
Grade 2	217 (65.0%)
Grade 3	44 (13.2%)
TNM stage	
Stage I	71 (21.2%)
Stage II	110 (32.8%)
Stage III	110 (32.8%)
Stage IV	44 (13.1%)
Mismatch repair (MMR) enzyme screening status	
Deficient	38 (11.3%)
Proficient	297 (88.7%)
Modified Glasgow Prognostic Score	
0	262 (78.4%)
1	64 (19.2%)
2	8 (2.4%)
Systemic inflammatory markers	
Serum C-reactive protein, mg/L, median (IQR)	2.80 (0.81–8.70)
Serum albumin, g/L, median (IQR)	43.0 (40.0–45.0)
Blood neutrophil to lymphocyte ratio, median (IQR)	2.42 (1.73–3.29)
Amino acids, µmol/L, mean (SD)	
Alanine	452.4 (89.1)
Glutamine	488.6 (87.0)
Glycine	282.1 (60.5)
Histidine	57.6 (10.6)
Isoleucine	55.6 (18.7)
Leucine	77.8 (24.3)
Valine	173.2 (49.0)
Phenylalanine	84.6 (18.4)
Tyrosine	57.4 (16.6)

*SD* standard deviation, *IQR* interquartile range

### Relationship of serum amino-acid levels and systemic inflammatory markers

Systemic inflammation is a characteristic feature of cancer cachexia. Therefore, we first assessed the relationships between serum AA levels and systemic inflammatory markers. Patients with systemic inflammation, as evidenced by increased mGPS, had lower glutamine (*p* < 0.001) and histidine levels (*p* < 0.001) and higher phenylalanine levels (*p* < 0.001) (Table 2).

**Table 2:** Serum amino-acid levels in relation to mGPS

	mGPS 0 (*n* = 262)	mGPS 1–2 (*n* = 72)		
	µmol/L, mean (SD)	µmol/L, mean (SD)	*p* value	Adjusted *p* value
Alanine	457.7 (90.7)	433.7 (81.5)	0.043	0.298
Glutamine	499.5 (79.8)	445.0 (97.7)	**4.0E-4**	**1.1E-4**
Glycine	279.2 (61.0)	290.4 (55.6)	0.162	0.598
Histidine	59.8 (9.8)	49.5 (9.7)	**3.3E-14**	**8.8E-11**
Isoleucine	56.5 (18.9)	52.9 (18.0)	0.144	0.321
Leucine	79.2 (24.0)	73.4 (24.3)	0.071	0.286
Valine	177.0 (47.9)	160.7 (50.6)	0.012	0.136
Phenylalanine	81.3 (13.5)	97.3 (26.7)	**4.0E-6**	**8.7E-10**
Tyrosine	58.2 (17.0)	54.9 (15.2)	0.133	0.414

*mGPS* modified Glasqow Prognostic Score, *SD* standard deviation. *p* values were adjusted for tumour location (colon vs. rectum), tumour stage variables (T1–2 vs. T3–4; N0 vs. N1–2; M0 vs. M1) and preoperative radiotherapy/chemoradiotherapy (no vs. yes). Significant *p* values are marked in bold

Of the components of mGPS, serum CRP negatively correlated with serum glutamine (*p* < 0.001) and histidine levels (*p* < 0.001) and positively correlated with serum phenylalanine levels (*p* < 0.001) (Table S2). Serum albumin levels positively correlated with serum levels of alanine (univariate *p* < 0.001; multivariate adjusted *p* = 0.002) and negatively correlated with serum phenylalanine levels (*p* < 0.001) (Table S2).

Increased neutrophil/lymphocyte ratio reflects systemic inflammation,^33^ and in our CRC cohort it was associated with lower serum histidine levels (univariate *p* < 0.001; multivariate adjusted *p* = 0.006) and higher serum phenylalanine levels (*p* = 0.001) (Table S2).

**Fig. 1:**
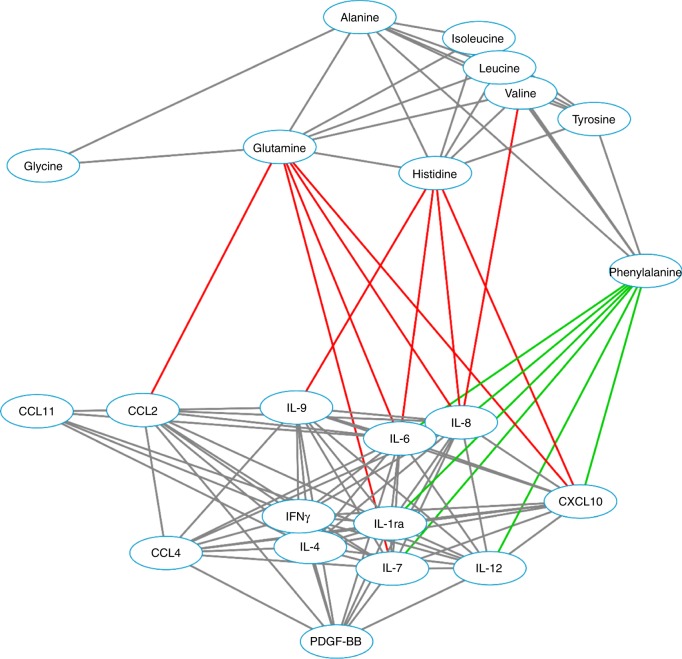
2D visualisation of the interrelationships between serum amino-acid levels and serum cytokine levels. Individual variables are represented by nodes and their associations are represented by edges (connecting lines). Only the associations with *p* < 0.01 are shown and the edge length illustrates the significance of the association. Grey edges show associations between two amino-acid variables or between two cytokine variables (all positive), whereas the correlations between amino acids and cytokines are represented by green (positive correlation) and red (negative correlation) edges

We correlated the serum AA levels with serum levels of 13 cytokines in 144/341 (42.2%) patients operated between April 2006 and January 2010. Figure 1 shows 2D visualisation of the interrelationships between serum AA levels and serum cytokines (Tables S3 and S4). Of these cytokines, pro-inflammatory cytokine IL-6 most strongly associated with increased phenylalanine levels (*p* < 0.001) and lower histidine levels (univariate *p* < 0.001; multivariate adjusted *p* = 0.003), whereas pro-inflammatory chemokine IL-8 associated with decreased glutamine levels (*p* < 0.001) and increased phenylalanine levels (univariate *p* < 0.001; multivariate adjusted *p* = 0.002). In addition, glutamine and histidine levels showed negative correlation with several other cytokines and phenylalanine positive correlations with multiple cytokines, including IL-1ra, IL-6, IL-7, IL-8, IL-12 and CXCL10. As expected, the AAs, which showed no statistically significant associations with mGPS, showed weaker associations with cytokine levels than glutamine, histidine and phenylalanine (Tables S3 and S4).

### Serum amino-acid levels in relation to clinicopathological characteristics of CRC

Next, we assessed the relationships between AA levels and the clinicopathological parameters. The results of the three AAs (glutamine, histidine, phenylalanine) that showed the strongest association with systemic inflammation are displayed in Table 3, whereas the relationships between other AAs and clinicopathological factors are portrayed in Tables S5 and S6.

**Table 3:** Associations of serum glutamine, histidine and phenylalanine levels with clinicopathological characteristics

	Glutamine		Histidine		Phenylalanine	
	µmol/L, mean (SD)	*p* value	µmol/L, mean (SD)	*p* value	µmol/L, mean (SD)	*p* value
Gender						
Male	490.9 (84.2)	0.597	59.1 (10.3)	**0.005**	84.6 (15.2)	0.920
Female	485.8 (90.4)		55.8 (10.7)		84.8 (21.5)	
Age						
< 65 years	475.6 (90.5)	0.039	59.5 (11.3)	0.014	83.0 (16.7)	0.202
≥ 65 years	496.0 (84.3)		56.5 (10.1)		85.6 (19.2)	
BMI (kg/m^2^)						
< 20	511.8 (89.9)	0.797	54.8 (8.8)	0.758	76.5 (14.3)	0.241
20–25	484.3 (96.3)		57.9 (10.8)		84.9 (16.3)	
25–30	491.3 (78.6)		57.7 (9.7)		84.1 (18.6)	
30–35	494.6 (92.7)		59.0 (12.5)		88.8 (24.3)	
≥ 35	482.0 (80.7)		58.1 (10.1)		84.3 (12.5)	
Tumour location						
Proximal	488.3 (95.1)	0.923	55.8 (10.4)	0.082	86.4 (20.6)	0.374
Distal	497.0 (95.2)		58.3 (12.3)		84.6 (16.3)	
Rectum	488.3 (75.3)		58.4 (9.4)		82.8 (13.1)	
Preoperative RT/CRT in rectal cancers						
No	497.4 (75.2)	0.077	57.8 (10.6)	0.192	82.6 (15.0)	0.616
Yes	475.4 (74.6)		59.9 (8.9)		83.8 (12.6)	
TNM stage						
Stage I	510.3 (86.7)	**0.002**	59.2 (8.9)	**0.001**	83.8 (22.1)	0.031
Stage II	479.4 (86.4)		56.6 (10.9)		83.1 (13.5)	
Stage III	503.0 (74.1)		60.0 (9.8)		84.7 (13.8)	
Stage IV	453.8 (100.7)		52.7 (9.4)		88.9 (20.4)	
Depth of invasion						
T1	519.7 (115.9)	0.023	59.2 (8.2)	0.022	80.2 (14.6)	0.252
T2	513.1 (76.3)		60.2 (9.3)		82.9 (21.2)	
T3	481.8 (84.3)		56.9 (10.5)		84.8 (14.5)	
T4	480.5 (103.2)		55.2 (9.9)		89.5 (20.0)	
Nodal metastasis						
N0	490.3 (88.8)	0.432	57.3 (10.4)	0.418	83.2 (17.6)	0.096
N1	500.7 (80.5)		58.7 (10.3)		85.3 (15.7)	
N2	475.8 (85.0)		57.1 (9.2)		87.4 (14.9)	
Distant metastasis						
M0	495.9 (82.9)	**0.009**	58.4 (10.1)	**0.005**	83.8 (16.1)	0.040
M1	453.8 (100.7)		52.7 (9.4)		88.9 (20.4)	
Lymphatic invasion						
No	489.7 (85.7)	0.842	57.8 (9.7)	0.778	84.0 (18.4)	0.454
Yes	487.8 (89.4)		57.5 (11.1)		85.5 (18.4)	
Blood vessel invasion						
No	492.3 (85.4)	0.096	58.0 (10.0)	0.134	83.5 (17.0)	0.027
Yes	470.3 (95.2)		55.7 (11.9)		91.0 (23.5)	
WHO grade 1–3						
Grade 1	511.0 (79.3)	0.013	58.1 (11.1)	0.089	84.4 (22.0)	0.660
Grade 2	488.7 (81.5)		58.0 (9.8)		84.0 (15.0)	
Grade 3	462.8 (112.9)		54.6 (11.1)		87.1 (14.4)	
MMR screening status						
MMR deficient	497.2 (112.0)	0.530	55.2 (12.2)	0.113	86.8 (27.6)	0.465
MMR proficient	487.7 (83.4)		58.0 (10.2)		84.4 (16.9)	
BRAF VE1 immunohistochemistry						
Negative	488.7 (86.9)	0.782	57.8 (10.6)	0.307	83.9 (16.9)	0.025
Positive	484.2 (90.1)		55.7 (11.1)		91.7 (28.8)	

*SD* standard deviation, *BMI* body mass index, *RT/CRT* radiotherapy/chemoradiotherapy, *MMR* mismatch repair. Significant *p* values are marked in bold.

Female patients had lower levels of histidine (*p* = 0.005), glycine (*p* < 0.001), isoleucine (*p* < 0.001), leucine (*p* < 0.001), and valine (*p* < 0.001). There were no statistically significant correlations between AA levels and patient age, body mass index, tumour location and neoadjuvant treatment.

TNM stage IV, i.e., presence of distant metastasis, associated with lower levels of glutamine (*p* = 0.002) and histidine (*p* = 0.001). Moreover, high tumour stage was associated with lower levels of alanine (*p* = 0.003) and higher glycine levels (*p* = 0.001). Higher T-class (depth of invasion) and N-class (nodal metastasis) were associated with increased glycine levels (*p* = 0.009 and *p* < 0.001). Distant metastasis was associated with decreased levels of glutamine (*p* = 0.009) and histidine (*p* = 0.005). Tumour differentiation did not show statistically significant correlations with AA levels.

CRCs developing from serrated precursor lesions have different histological, molecular, and aetiological features than other CRCs.^34^ Therefore, we hypothesised that these differences could be reflected in serum AA profile. However, we could not observe any statistically significant associations between serum AA levels and BRAF mutation and MMR deficiency, which are characteristic molecular features of the serrated pathway of CRC.^34^

The high proliferation rate of cancer cells creates demand for ATP, lipids, nucleotides, and proteins. Thus, we examined serum AA levels in relation to tumour proliferation assessed by Ki-67 score. However, none of the AAs analysed associated with tumour proliferation (Table S4).

As AAs serve as nutrients for tumour-associated immune cells, we investigated the correlations between serum AA levels and the densities of five types of tumour infiltrating immune cells (Table S7). However, no statistically significant correlations were observed.

### Relationship of serum amino-acid levels and systemic inflammatory markers in CRC patients with local or metastasised disease

As cancer dissemination may induce metabolic alterations, we did subgroup analyses to enlighten the individual contribution of cancer stage and systemic inflammation to serum AA levels. First, we analysed serum AA levels in relation to systemic inflammation in CRC patients with local disease (stages 1–3, Table S8). As in the whole CRC cohort, CRC patients with stage I–III disease and systemic inflammation (elevated mGPS) had lower glutamine (*p* = 0.008) and histidine levels (*p* < 0.001) and higher phenylalanine levels (*p* < 0.001) than those stage I–III patients who did not have elevated mGPS. Interestingly, also valine levels were decreased in CRC patients with stage I–III disease and activated systemic inflammation.

Next, we analysed CRC patients with distant metastases (stage IV), and also in these patients, elevated mGPS was associated with decreased glutamine and histidine levels and increased phenylalanine levels (Table S9).

When we analysed patients without laboratory evidence of systemic inflammation (mGPS = 0), the presence of distant metastasis did not associate with serum glutamine (*p* = 0.540), histidine (*p* = 0.146) and phenylalanine (*p* = 0.112) levels. This suggests that systemic inflammation was more important determinant of serum glutamine, histidine and phenylalanine levels than tumour stage. In contrast, serum valine levels were low in all CRC patients with distant metastasis regardless of their mGPS.

### Multivariate analyses

To identify independent associations between serum AA levels and factors showing associations in univariate analysis, we performed multiple linear regression analyses (Table 4 and S10). Factors analysed were gender, invasion through muscularis propria, nodal metastasis, distant metastasis and activation of systemic inflammation (mGPS). In these analyses, as well, the most prominent indicator of serum glutamine, histidine and phenylalanine levels was mGPS. Gender was the main predictor of serum glycine and valine levels.

**Table 4:** Multiple linear regression model of glutamine levels in colorectal cancer patients

Amino acid	Independent	Beta	*p* value
Glutamine	Model 1		
	mGPS	−0.244	**7.0E-6**
	Model 2		
	mGPS	−0.225	**3.5E-5**
	Invasion through muscularis propria	−0.140	**0.009**

*mGPS* modified Glasqow Prognostic Score. Significant *p* values are marked in bold

### Survival analyses

**Fig. 2:**
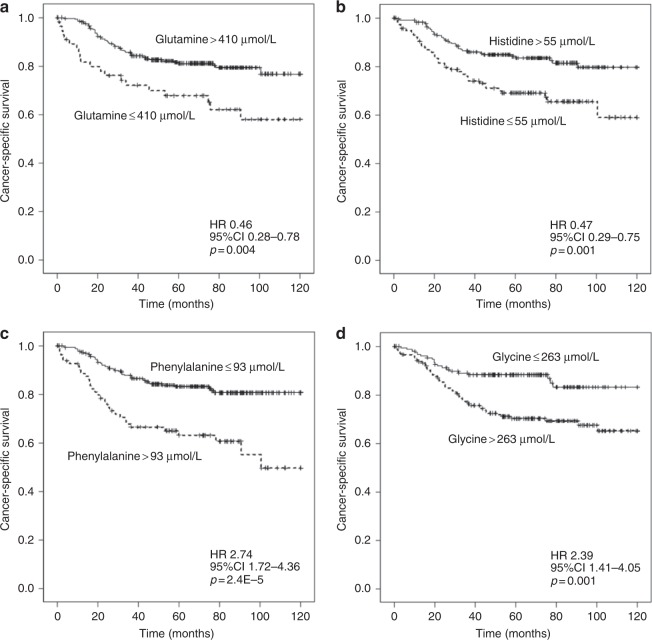
Kaplan–Meier survival curves for cancer-specific survival of patients with low and high serum levels of glutamine **a**, histidine **b**, phenylalanine **c** and glycine **d**

Finally, we studied the prognostic value of serum AA levels in 120-month follow-up (Fig. 2, Tables S11–S16). Table S11 shows the ROC analysis area under the curves and cutoff points. High serum histidine level was associated with better CSS (*p* = 0.001) and OS (*p* = 0.001) and increased glutamine with better CSS (*p* = 0.004) and OS (*p* = 0.005). High phenylalanine level was associated with decreased CSS (*p* < 0.001) and OS (*p* < 0.001) and high glycine level with worse CSS (*p* = 0.001). As glutamine, histidine, phenylalanine and glycine showed significant association with survival in the univariate analyses, they were included in the multivariate survival models (Tables S13–S16). However, in these models, none of these AAs significantly associated with CSS.

## Discussion

In this study, we analysed the factors contributing to serum AA levels in CRC, focusing on the impact of systemic inflammation on the serum AA profile. We found decreased serum glutamine and histidine levels and increased phenylalanine levels in patients with systemic inflammation. In addition, high tumour stage was associated with lower alanine and higher glycine levels. These findings indicate that the AA serum profile in CRC was determined by both systemic inflammation and tumour characteristics, mainly tumour stage.

Glutamine is an important inter-organ carbon and nitrogen transporter, and the main source of circulating glutamine is skeletal muscle.^13^ In healthy humans, glutamine is mainly consumed in the gut and kidney.^13^ Tumour tissue can either consume or produce glutamine depending on tissue of origin and oncogene activation.^35^ Our study indicated that the presence of systemic inflammation is the main indicator of decreased glutamine levels in CRC patients. Immune cells consume glutamine,^13^ and decreased plasma glutamine levels have been reported in patients with sepsis and in critically ill patients on ICU admission.^36,37^ In sepsis patients, lower glutamine plasma levels result from increased glutamine consumption.^38^ We found decreased glutamine levels in our patients with systemic inflammation, suggesting that glutamine production does not match its requirements when systemic inflammation is present. Glutaminolysis, as well as aerobic glycolysis, is extensively increased in T cells upon activation.^39,40^ Thus, lymphocyte activation during systemic inflammation could contribute to the increased glutamine consumption. Another AA consumer during systemic inflammation is the liver, where AAs are consumed in gluconeogenesis and also utilised to produce acute phase response proteins.^41^ However, alanine is the major gluconeogenic AA and protein synthesis requires all AAs. Thus, inflammatory cell activation may be a major glutamine-consuming cascade during systemic inflammation.

We found a strong negative correlation between serum levels of glutamine and CRP, IL-6 and IL-8 (Fig. 1, Tables S4 and S5). Tumour-induced IL-6 is considered an essential player in cancer-associated metabolic alterations,^42^ and IL-6 is able to induce muscle and fat wasting in mice.^43,44^ Interestingly, IL-6 alters AA turnover; it reduces the arterial AA concentrations albeit the net muscle protein breakdown is increased.^45^ This suggests that the IL-6 could increase the demand of glutamine in CRC patients with systemic inflammation. In regard to the other analysed AAs, only histidine was significantly negatively correlated with serum IL-6. Histidine is an essential AA synthesised to histamine by the histidine decarboxylase (HDC) enzyme. Histamine regulates various physiological and pathophysiological processes, including allergic reactions, gastric acid secretion, neurotransmission and immune responses. In CRC, increased HDC activity and histamine level in the neoplastic tissue have been shown to correlate with tumour progression.^46^ Mast cells and basophils are the main cells that synthetise and store histamine. IL-6 has been shown to increase mast cell numbers and reactivity.^47^ Thus, we speculate that the negative correlation between IL-6 and histidine levels may reflect increased consumption of histidine in histamine synthesis during systemic inflammation. Our observations of the AA profile in CRC suggest that muscle protein breakdown is accelerated during systemic inflammation, supporting the role of systemic inflammation in cancer cachexia pathogenesis. However, other mechanisms of cachexia might exist in other cancer types. For example, in pancreatic cancer, whole-body protein breakdown is an early event in cancer development, and blood glutamine levels decrease along pancreatic cancer onset and progression.^48,49^

Contrary to serum glutamine and histidine levels, we found increased phenylalanine levels in CRC patients with systemic inflammation. In phenylalanine catabolism, phenylalanine is first metabolised to tyrosine by phenylalanine hydroxylase (PAH) in the liver. Increased blood concentrations of phenylalanine have been described in inflammatory conditions such as HIV-1 infection,^50^ sepsis^51^ and cancer.^52^ The reason for this is unclear, but might be related to extensive changes in gene expression in liver during systemic inflammation and/or to oxidative stress resulting from chronic inflammation. In order to function properly, PAH requires the cofactor 5,6,7,8-tetrahydrobiopterin (BH4), which is highly sensitive to oxidation. The clinical consequences of increased phenylalanine level in CRC patients are also not clear. However, the concentrations that we observed (highest 231 µmol/L) are not toxic, as optimal serum phenylalanine level for patients with phenylketonuria is 500 µmol/L or less.^53^

In this study, female sex was the main indicator of high serum glycine, and low valine, isoleucine and leucine levels in CRC patients. Serum histidine levels were also determined by gender, but systemic inflammation was the main determinant of histidine levels. Earlier study on healthy subjects has shown gender-related differences in serum AA levels, and similar to CRC patients, healthy women had increased glycine but decreased histidine, isoleucine, leucine, phenylalanine, tyrosine and valine levels.^54^ However, unlike in healthy subjects, in CRC patients serum phenylalanine and tyrosine levels did not show significant association with gender. In CRC patients, serum phenylalanine levels showed strong association with systemic inflammation potentially masking less prominent associations with gender.

Improved prognostic and predictive parameters would help to better classify CRC patients into therapeutically relevant groups. In our univariate survival analyses, high serum levels of histidine and glutamine associated with better CSS, whereas high phenylalanine and glycine levels associated with worse CSS. However, none of these associations was significant in multivariate Cox regression models. One limitation of our study in this regard was that our material was based on unselected stage I–IV CRC patients. Therefore, further studies are required to assess, whether serum AAs could predict survival in more specifically defined patient subgroups such as stage II patients. Owing to the sample size, such subgroup analyses with adequate statistical power were not considered possible in this study. Further studies should also evaluate, whether the adverse outcome associated with serum AA level alterations or cancer-associated cachexia could be reversed with AA supplementation or treatment for systemic inflammation.

Several limitations of our study need to be considered. Multiple processes contribute to the circulating metabolomic profile, including the effects of the tumour and the inflammatory cells, the patients’ diet and lifestyle, as well as other environmental exposures. Although we extensively analysed the circulating inflammatory markers, we did not survey specific lifestyle or diet data, which could confound the results. Loss of appetite is common phenomenon in many patients with cancer. However, muscle wasting is resistant to conventional nutritional support suggesting minor effect of diet on our results.^2,3^ Moreover, multiple hypotheses were tested in this observational study. To reduce the risk of type 1 statistical error, we decided to lower the threshold of statistical significance to *p* < 0.01. However, this approach results in some increase of type 2 error. The major advantage of our study is the prospectively recruited patient material with extensive and standardised histopathological analysis, analysis of multiple AAs, multiple systemic inflammatory markers and tumour infiltrating immune cell types, which extends the perspective of the results relative to analysis of a single marker.

In conclusion, serum AA levels in CRC reflect systemic inflammation and disease stage. Particularly, low serum histidine and glutamine levels and high serum phenylalanine levels were associated with systemic inflammation. Our studies together with previous observations suggest that systemic inflammation is associated with enhanced glutamine consumption and muscle wasting, supporting the role of systemic inflammation in cancer cachexia pathogenesis. Further investigations are needed to define the roles of serum AAs as markers for prognosis, cachexia and possible targets for intervention in CRC.

## Electronic supplementary material

Supplementary tables 1–16

Supplementary Figure S1

## **Data availability**

The data sets generated and analysed during the current study are available from the corresponding author on reasonable request.
