# Supplementary material for: Alterations in serum amino-acid profile in the progression of colorectal cancer: associations with systemic inflammation, tumour stage and patient survival
Source: Br J Cancer. 2018 Dec 19;120(2):238–46. doi: 10.1038/s41416-018-0357-6 (PMC6342921; doi:10.1038/s41416-018-0357-6)
Supplement: Supplementary file 1 — Supplementary tables 1–16 [file 41416_2018_357_MOESM1_ESM.docx]

**Supplementary online material**

**Table S1**. The laboratory reference intervals for serum amino acids.

| **Amino acids, µmol/L, mean (SD)** | **Measured, all patients** | **Measured, patients with elevated mGPS** | **Reference value** | **Number of patients with aa level outside the range** |
| --- | --- | --- | --- | --- |
| Alanine | 452.4 (89.1) | 433.7 (81.5) | 210-661 | 0 under, 9 over (2.7%) |
| Glutamine | 488.6 (87.0) | 445.0 (97.7) | 420-700 | 66 under, 0 over (19.8%) |
| Glycine | 282.1 (60.5) | 290.4 (55.6) | 120-560 | 0 under, 0 over |
| Histidine | 57.6 (10.6) | 49.5 (9.7) | 32-110 | 4 under, 0 over (1.2%) |
| Isoleucine | 55.6 (18.7) | 52.9 (18.0) | 40-100 | 67 under (20.0%), 12 over (3.6%) |
| Leucine | 77.8 (24.3) | 73.4 (24.3) | 75-175 | 172 under (51.3%), 0 over |
| Valine | 173.2 (49.0) | 160.7 (50.6) | 145-315 | 101 under (30.1%), 5 over (1.5%) |
| Phenylalanine | 84.6 (18.4) | 97.3 (26.7) | 35-90 | 0 under, 96 over (28.6%) |
| Tyrosine | 57.4 (16.6) | 54.9 (15.2) | 20-90 | 1 under (0.3%), 15 over (4.5%) |

Abbreviations: mGPS: modified Glasgow Prognostic score: SD: standard deviation; aa: amino acid.

**Table S2.** Correlations between serum alanine, glycine, isoleucine, leucine, valine and tyrosine levels and peripheral blood cell counts, the NLR, CRP and albumin

|  |  |  | **NLR** | **Leukocytes** | **Neutrophils** | **Lymphocytes** | **Monocytes** | **Eosinophils** | **Basophils** | **Serum CRP** | **Serum albumin** |
| --- | --- | --- | --- | --- | --- | --- | --- | --- | --- | --- | --- |
| **Gln** | Unadjusted | Pearson r | -0.214 | -0.114 | -0.191 | 0.070 | -0.110 | 0.034 | 0.133 | -0.300 | 0.044 |
|  |  | p value | **9.5E-5** | 0.037 | **4.9E-4** | 0.205 | 0.047 | 0.535 | 0.016 | **3.0E-8** | 0.422 |
|  | Adjusted | Beta | -0.145 | -0.101 | -0.171 | 0.009 | -0.121 | 0.052 | 0.088 | -0.260 | 0.006 |
|  |  | p value | 0.011 | 0.084 | **0.003** | 0.894 | 0.033 | 0.343 | 0.119 | **3.0E-6** | 0.911 |
| **His** | Unadjusted | Pearson r | -0.202 | -0.193 | -0.227 | 0.023 | -0.170 | 0.008 | 0.041 | -0.420 | 0.190 |
|  |  | p value | **2.2E-4** | **3.7E-4** | **3.1E-5** | 0.682 | **0.002** | 0.879 | 0.462 | **1.5E-15** | **4.6E-4** |
|  | Adjusted | Beta | -0.153 | -0.099 | -0.133 | 0.068 | -0.134 | 0.001 | 0.018 | -0.388 | 0.138 |
|  |  | p value | **0.006** | 0.092 | 0.021 | 0.300 | 0.019 | 0.989 | 0.748 | **1.2E-12** | 0.011 |
| **Phe** | Unadjusted | Pearson r | 0.175 | 0.203 | 0.266 | 0.045 | 0.243 | 0.025 | 0.06 | 0.416 | -0.211 |
|  |  | p value | **0.001** | **1.9E-4** | **8.4E-7** | 0.418 | **7.7E-6** | 0.645 | 0.277 | **2.7E-15** | **9.9E-5** |
|  | Adjusted | Beta | 0.191 | 0.207 | 0.270 | 0.041 | 0.232 | 0.018 | 0.048 | 0.406 | -0.194 |
|  |  | p value | **0.001** | **4.5E-4** | **2.0E-6** | 0.533 | **4.8E-5** | 0.749 | 0.397 | **1.9E-13** | **3.5E-4** |
| **Ala** | Unadjusted | Pearson r | -0.044 | 0.079 | 0.040 | 0.088 | 0.035 | 0.100 | 0.130 | -0.084 | 0.200 |
|  |  | p value | 0.423 | 0.151 | 0.466 | 0.110 | 0.524 | 0.068 | 0.018 | 0.125 | **2.2E-04** |
|  | Adjusted | Beta | 0.030 | 0.151 | 0.115 | 0.090 | 0.059 | 0.110 | 0.103 | -0.030 | 0.169 |
|  |  | p value | 0.611 | 0.011 | 0.050 | 0.174 | 0.309 | 0.047 | 0.074 | 0.604 | **0.002** |
| **Gly** | Unadjusted | Pearson r | 0.029 | 0.063 | 0.012 | -0.023 | -0.020 | 0.127 | -0.009 | 0.045 | -0.039 |
|  |  | p value | 0.600 | 0.259 | 0.836 | 0.682 | 0.726 | 0.023 | 0.879 | 0.423 | 0.481 |
|  | Adjusted | Beta | 0.007 | 0.056 | -0.006 | -0.017 | -0.053 | 0.102 | -0.010 | -0.019 | -0.011 |
|  |  | p value | 0.900 | 0.354 | 0.924 | 0.796 | 0.360 | 0.064 | 0.857 | 0.737 | 0.846 |
| **Ile** | Unadjusted | Pearson r | -0.044 | 0.031 | 0.037 | 0.085 | 0.001 | 0.133 | 0.033 | -0.036 | 0.074 |
|  |  | p value | 0.421 | 0.568 | 0.507 | 0.124 | 0.980 | 0.015 | 0.556 | 0.520 | 0.177 |
|  | Adjusted | Beta | -0.028 | 0.085 | 0.089 | 0.144 | 0.033 | 0.136 | 0.024 | -0.008 | 0.051 |
|  |  | p value | 0.633 | 0.160 | 0.134 | 0.031 | 0.578 | 0.015 | 0.682 | 0.892 | 0.360 |
| **Leu** | Unadjusted | Pearson r | -0.002 | -0.022 | 0.020 | 0.021 | -0.023 | 0.092 | -0.014 | -0.020 | 0.153 |
|  |  | p value | 0.969 | 0.683 | 0.716 | 0.705 | 0.681 | 0.095 | 0.802 | 0.720 | **0.005** |
|  | Adjusted | Beta | 0.005 | 0.070 | 0.109 | 0.120 | 0.035 | 0.087 | 0.001 | 0.029 | 0.133 |
|  |  | p value | 0.930 | 0.248 | 0.063 | 0.073 | 0.547 | 0.118 | 0.986 | 0.616 | 0.016 |
| **Val** | Unadjusted | Pearson r | -0.031 | -0.074 | -0.031 | 0.007 | -0.056 | 0.104 | -0.060 | -0.048 | 0.148 |
|  |  | p value | 0.576 | 0.179 | 0.573 | 0.901 | 0.309 | 0.059 | 0.279 | 0.380 | **0.007** |
|  | Adjusted | Beta | -0.025 | 0.031 | 0.071 | 0.119 | 0.013 | 0.097 | -0.046 | 0.015 | 0.118 |
|  |  | p value | 0.667 | 0.602 | 0.227 | 0.072 | 0.819 | 0.077 | 0.423 | 0.801 | 0.032 |
| **Tyr** | Unadjusted | Pearson r | 0.001 | 0.040 | 0.030 | 0.027 | 0.090 | 0.100 | 0.050 | 0.063 | 0.081 |
|  |  | p value | 0.991 | 0.461 | 0.584 | 0.625 | 0.104 | 0.069 | 0.363 | 0.254 | 0.140 |
|  | Adjusted | Beta | 0.043 | 0.100 | 0.087 | 0.038 | 0.125 | 0.104 | 0.037 | 0.115 | 0.063 |
|  |  | p value | 0.460 | 0.096 | 0.139 | 0.568 | 0.031 | 0.061 | 0.527 | 0.048 | 0.259 |

The correlations were adjusted for tumor location (colon vs. rectum), tumor stage variables (T1-2 vs. T3-4; N0 vs. N1-2; M0 vs. M1) and preoperative radiotherapy/chemoradiotherapy (no vs. yes). Abbreviations: NLR: neutrophil/lymphocyte ratio; CRP: C-reactive protein; Gln: glutamine; His: histidine; Phe: phenylalanine; Ala: alanine; Gly: glycine; Ile: isoleucine; Leu: leucine, Val: valine; Tyr: tyrosine. NLR, peripheral blood cell counts and serum CRP were logarithmically transformed.

**Table S3.** Correlations between serum amino acid levels and serum cytokine levels

|  |  |  | IL-1ra | IL-4 | IL-6 | IL-7 | IL-8 | IL-9 | IL-12 |
| --- | --- | --- | --- | --- | --- | --- | --- | --- | --- |
| Glutamine | Unadjusted | Pearson r | -0.161 | -0.071 | -0.266 | -0.230 | -0.324 | -0.215 | -0.097 |
|  |  | p value | 0.057 | 0.400 | **0.002** | **1.4E-4** | **8.7E-5** | 0.011 | 0.250 |
|  | Adjusted | Beta | -0.086 | -0.042 | -0.199 | -0.177 | -0.341 | -0.192 | -0.064 |
|  |  | p value | 0.327 | 0.620 | 0.021 | 0.040 | **3.9E-4** | 0.023 | 0.449 |
| Histidine | Unadjusted | Pearson r | -0.205 | -0.103 | -0.396 | -0.126 | -0.303 | -0.257 | -0.158 |
|  |  | p value | 0.014 | 0.220 | **9.5E-7** | 0.131 | **2.3E-4** | **0.002** | 0.058 |
|  | Adjusted | Beta | -0.075 | -0.015 | -0.252 | -0.054 | -0.226 | -0.163 | -0.079 |
|  |  | p value | 0.389 | 0.856 | **0.003** | 0.529 | 0.023 | 0.052 | 0.347 |
| Phenylalanine | Unadjusted | Pearson r | 0.267 | 0.102 | 0.318 | 0.260 | 0.312 | 0.097 | 0.334 |
|  |  | p value | **0.001** | 0.223 | **1.1E-4** | **0.002** | **1.4E-4** | 0.247 | **4.4E-5** |
|  | Adjusted | Beta | 0.246 | 0.032 | 0.336 | 0.246 | 0.316 | 0.093 | 0.332 |
|  |  | p value | **0.005** | 0.754 | **1.2E-4** | **0.005** | **0.002** | 0.280 | **6.8E-5** |
| Alanine | Unadjusted | Pearson r | 0.019 | -0.034 | -0.063 | 0.013 | -0.085 | -0.095 | 0.045 |
|  |  | p value | 0.819 | 0.688 | 0.454 | 0.879 | 0.313 | 0.261 | 0.590 |
|  | Adjusted | Beta | 0.120 | 0.015 | 0.070 | 0.080 | -0.020 | -0.023 | 0.096 |
|  |  | p value | 0.178 | 0.862 | 0.430 | 0.357 | 0.846 | 0.786 | 0.256 |
| Glycine | Unadjusted | Pearson r | 0.033 | 0.009 | 0.023 | 0.000 | 0.234 | -0.041 | 0.086 |
|  |  | p value | 0.703 | 0.913 | 0.794 | 0.999 | **0.006** | 0.637 | 0.314 |
|  | Adjusted | Beta | -0.021 | -0.011 | -0.020 | -0.029 | 0.175 | -0.046 | 0.069 |
|  |  | p value | 0.817 | 0.894 | 0.822 | 0.738 | 0.085 | 0.591 | 0.419 |
| Isoleucine | Unadjusted | Pearson r | -0.083 | -0.142 | -0.076 | -0.008 | -0.091 | -0.237 | -0.055 |
|  |  | p value | 0.324 | 0.090 | 0.371 | 0.928 | 0.282 | **0.004** | 0.518 |
|  | Adjusted | Beta | -0.035 | -0.115 | 0.021 | 0.018 | -0.035 | -0.205 | -0.020 |
|  |  | p value | 0.700 | 0.191 | 0.814 | 0.838 | 0.736 | 0.019 | 0.819 |
| Leucine | Unadjusted | Pearson r | -0.100 | -0.187 | -0.109 | -0.011 | -0.157 | -0.202 | -0.003 |
|  |  | p value | 0.233 | 0.026 | 0.195 | 0.892 | 0.061 | 0.016 | 0.970 |
|  | Adjusted | Beta | -0.021 | -0.136 | 0.021 | 0.034 | -0.053 | -0.136 | 0.062 |
|  |  | p value | 0.810 | 0.113 | 0.813 | 0.698 | 0.601 | 0.113 | 0.466 |
| Valine | Unadjusted | Pearson r | -0.166 | -0.255 | -0.179 | -0.055 | -0.231 | -0.190 | -0.001 |
|  |  | p value | 0.048 | **0.002** | 0.033 | 0.517 | 0.005 | 0.024 | 0.986 |
|  | Adjusted | Beta | -0.079 | -0.196 | -0.048 | -0.006 | -0.126 | -0.110 | 0.073 |
|  |  | p value | 0.368 | 0.021 | 0.587 | 0.944 | 0.212 | 0.195 | 0.385 |
| Tyrosine | Unadjusted | Pearson r | -0.084 | -0.196 | -0.052 | -0.035 | -0.152 | -0.122 | 0.090 |
|  |  | p value | 0.315 | 0.019 | 0.535 | 0.679 | 0.069 | 0.145 | 0.286 |
|  | Adjusted | Beta | -0.060 | -0.182 | 0.015 | -0.008 | -0.148 | -0.084 | 0.115 |
|  |  | p value | 0.505 | 0.037 | 0.868 | 0.929 | 0.150 | 0.339 | 0.182 |

The correlations were adjusted for tumor location (colon vs. rectum), tumor stage variables (T1-2 vs. T3-4; N0 vs. N1-2; M0 vs. M1) and preoperative radiotherapy/chemoradiotherapy (no vs. yes). Abbreviations: IL: interleukin. Serum cytokine levels were logarithmically transformed.

**Table S4.** Correlations between serum amino acid levels and serum cytokine levels and Ki-67

|  |  |  | IFNγ | CXCL10 | CCL2 | CCL4 | CCL11 | PDGF-BB | Ki-67 |
| --- | --- | --- | --- | --- | --- | --- | --- | --- | --- |
| Glutamine | Unadjusted | Pearson r | -0.115 | -0.217 | -0.221 | -0.151 | 0.111 | -0.062 | 0.145 |
|  |  | p value | 0.174 | 0.010 | **0.009** | 0.073 | 0.190 | 0.464 | 0.092 |
|  | Adjusted | Beta | -0.099 | -0.176 | -0.179 | -0.126 | 0.098 | -0.004 | 0.121 |
|  |  | p value | 0.243 | 0.038 | 0.037 | 0.133 | 0.248 | 0.960 | 0.161 |
| Histidine | Unadjusted | Pearson r | -0.132 | -0.303 | -0.033 | -0.122 | -0.047 | -0.076 | 0.002 |
|  |  | p value | 0.116 | **2.2E-4** | 0.692 | 0.144 | 0.572 | 0.366 | 0.986 |
|  | Adjusted | Beta | -0.055 | -0.261 | 0.055 | -0.094 | -0.101 | 0.015 | 0.021 |
|  |  | p value | 0.515 | **0.002** | 0.520 | 0.256 | 0.227 | 0.859 | 0.800 |
| Phenylalanine | Unadjusted | Pearson r | 0.141 | 0.290 | 0.155 | -0.035 | 0.084 | 0.028 | 0.032 |
|  |  | p value | 0.092 | **4.3E-4** | 0.064 | 0.674 | 0.318 | 0.737 | 0.708 |
|  | Adjusted | Beta | 0.125 | 0.282 | 0.135 | -0.029 | 0.113 | -0.003 | 0.064 |
|  |  | p value | 0.145 | **0.001** | 0.122 | 0.730 | 0.185 | 0.974 | 0.457 |
| Alanine | Unadjusted | Pearson r | -0.010 | 0.026 | -0.023 | -0.019 | 0.067 | -0.022 | 0.071 |
|  |  | p value | 0.904 | 0.759 | 0.785 | 0.823 | 0.425 | 0.791 | 0.406 |
|  | Adjusted | Beta | 0.036 | 0.092 | 0.046 | 0.015 | 0.066 | 0.050 | 0.075 |
|  |  | p value | 0.675 | 0.281 | 0.598 | 0.855 | 0.434 | 0.570 | 0.377 |
| Glycine | Unadjusted | Pearson r | -0.024 | 0.073 | 0.000 | 0.099 | -0.105 | 0.089 | 0.099 |
|  |  | p value | 0.783 | 0.395 | 0.997 | 0.246 | 0.219 | 0.299 | 0.253 |
|  | Adjusted | Beta | -0.035 | 0.070 | -0.020 | 0.097 | -0.074 | 0.082 | 0.126 |
|  |  | p value | 0.683 | 0.409 | 0.814 | 0.246 | 0.383 | 0.350 | 0.136 |
| Isoleucine | Unadjusted | Pearson r | -0.094 | -0.080 | -0.014 | -0.137 | -0.050 | -0.022 | -0.018 |
|  |  | p value | 0.267 | 0.340 | 0.867 | 0.103 | 0.557 | 0.793 | 0.834 |
|  | Adjusted | Beta | -0.064 | -0.048 | 0.029 | -0.132 | -0.083 | 0.009 | -0.004 |
|  |  | p value | 0.466 | 0.581 | 0.745 | 0.122 | 0.341 | 0.917 | 0.963 |
| Leucine | Unadjusted | Pearson r | -0.137 | -0.106 | -0.033 | -0.134 | -0.051 | -0.062 | 0.026 |
|  |  | p value | 0.102 | 0.206 | 0.696 | 0.111 | 0.546 | 0.459 | 0.758 |
|  | Adjusted | Beta | -0.084 | -0.063 | 0.010 | -0.127 | -0.078 | -0.013 | 0.040 |
|  |  | p value | 0.324 | 0.461 | 0.904 | 0.131 | 0.359 | 0.879 | 0.645 |
| Valine | Unadjusted | Pearson r | -0.184 | -0.109 | -0.088 | -0.141 | -0.048 | -0.122 | 0.007 |
|  |  | p value | 0.028 | 0.193 | 0.298 | 0.093 | 0.566 | 0.146 | 0.938 |
|  | Adjusted | Beta | -0.118 | -0.068 | -0.052 | -0.137 | -0.079 | -0.072 | 0.017 |
|  |  | p value | 0.163 | 0.422 | 0.546 | 0.099 | 0.347 | 0.409 | 0.838 |
| Tyrosine | Unadjusted | Pearson r | -0.121 | 0.087 | -0.121 | -0.131 | -0.028 | -0.174 | 0.039 |
|  |  | p value | 0.150 | 0.300 | 0.151 | 0.117 | 0.740 | 0.037 | 0.643 |
|  | Adjusted | Beta | -0.102 | 0.134 | -0.084 | -0.120 | -0.029 | -0.155 | 0.046 |
|  |  | p value | 0.239 | 0.122 | 0.341 | 0.158 | 0.734 | 0.081 | 0.601 |

| The correlations were adjusted for tumor location (colon vs. rectum), tumor stage variables (T1-2 vs. T3-4; N0 vs. N1-2; M0 vs. M1) and preoperative radiotherapy/chemoradiotherapy (no vs. yes). Abbreviations: IFN: interferon; CCL: chemokine (C-C motif) ligand; PDGF: platelet-derived growth factor. Serum cytokines and Ki-67 were logarithmically transformed. |
| --- |

**Table S5.** Associations of serum alanine, glycine and isoleucine levels with clinicopathological characteristics

|  | **Alanine** | | **Glycine** | | **Isoleucine** | |
| --- | --- | --- | --- | --- | --- | --- |
|  | **µmol/L, mean (SD)** | **p value** | **µmol/L, mean (SD)** | **p value** | **µmol/L, mean (SD)** | **p value** |
| **Gender** |  |  |  |  |  |  |
| Male  Female | 455.4 (88.7)  448.9 (89.7) | 0.502 | 266.5 (45.6)  299.8 (69.8) | **9.2E-7** | 60.1 (20.0)  50.6 (15.8) | **2.0E-6** |
| **Age** |  |  |  |  |  |  |
| < 65 years  ≥ 65 years | 450.7 (93.5)  453.3 (86.7) | 0.791 | 283.3 (63.7)  281.1 (58.7) | 0.791 | 55.4 (18.7)  55.8 (18.8) | 0.878 |
| **BMI (kg/m^2^)** |  |  |  |  |  |  |
| <20  20-25  25-30  30-35  ≥35 | 409.1 (65.8)  453.6 (97.9)  454.1 (78.1)  442.9 (91.7)  484.2 (114.4) | 0.148 | 307.1 (68.7)  293.4 (65.3)  276.9 (58.0)  277.6 (55.9)  272.6 (50.8) | 0.106 | 44.1 (17.4)  55.1 (20.3)  56.9 (18.5)  58.6 (17.1)  54.0 (17.1) | 0.119 |
| **Tumor location** |  |  |  |  |  |  |
| Proximal  Distal  Rectum | 452.2 (101.3)  459.3 (82.7)  451.5 (82.6) | 0.802 | 288.4 (63.1)  274.0 (49.5)  280.2 (62.1) | 0.364 | 56.0 (17.5)  56.3 (19.1)  55.1 (19.0) | 0.978 |
| **Preoperative RT/CRT in rectal cancers** | | |  |  |  |  |
| No  Yes | 450.7 (80.1)  451.4 (84.5) | 0.963 | 276.2 (64.1)  284.1 (59.8) | 0.443 | 54.0 (18.9)  57.3 (19.9) | 0.313 |
| **TNM Stage** |  |  |  |  |  |  |
| Stage I  Stage II  Stage III  Stage IV | 475.5 (77.0)  444.5 (102.1)  463.1 (84.9)  419.2 (65.1) | **0.003** | 268.4 (49.7)  272.6 (54.7)  290.7 (61.7)  308.5 (77.3) | **0.001** | 54.6 (15.9)  57.3 (20.1)  56.9 (18.3)  50.9 (16.9) | 0.296 |
| **Depth of invasion** |  |  |  |  |  |  |
| T1  T2  T3  T4 | 448.4 (81.8)  473.8 (79.6)  449.6 (93.6)  433.8 (67.3) | 0.527 | 248.8 (50.9)  279.0 (57.2)  281.1 (56.7)  317.6 (80.8) | **0.009** | 54.5 (20.3)  54.9 (16.0)  56.7 (19.1)  51.4 (17.9) | 0.410 |
| **Nodal metastasis** |  |  |  |  |  |  |
| N0  N1  N2 | 454.0 (93.7)  448.1 (80.5)  465.7 (84.2) | 0.518 | 271.2 (54.0)  287.7 (62.6)  311.0 (68.5) | **5.8E-5** | 56.1 (18.5)  55.4 (18.5)  55.5 (17.6) | 0.873 |
| **Distant metastasis** |  |  |  |  |  |  |
| M0  M1 | 459.1 (90.6)  419.2 (65.1) | 0.016 | 278.3 (56.9)  308.5 (77.3) | 0.014 | 56.5 (18.5)  50.9 (16.9) | 0.153 |
| **Lymphatic invasion** |  |  |  |  |  |  |
| No  Yes | 459.9 (95.3)  445.1 (80.1) | 0.129 | 272.9 (54.2)  290.7 (64.2) | **0.007** | 57.4 (19.5)  53.9 (17.4) | 0.092 |
| **Blood vessel invasion** |  |  |  |  |  |  |
| No  Yes | 453.0 (92.3)  453.9 (69.4) | 0.941 | 276.6 (56.6)  304.0 (69.0) | **0.002** | 56.4 (19.0)  52.7 (16.2) | 0.187 |
| **WHO Grade 1-3** |  |  |  |  |  |  |
| Grade 1  Grade 2  Grade 3 | 452.0 (81.4)  453.6 (87.4)  458.5 (109.7) | 0.862 | 278.8 (56.2)  280.5 (61.8)  294.2 (60.6) | 0.297 | 57.4 (20.5)  55.6 (17.8)  53.2 (17.0) | 0.338 |
| **MMR screening status** |  |  |  |  |  |  |
| MMR deficient  MMR proficient | 450.1 (119.9)  453.3 (84.1) | 0.835 | 276.3 (54.5)  282.8 (61.3) | 0.540 | 58.4 (19.1)  55.4 (18.6) | 0.359 |
| **BRAF VE1 immunohistochemistry** | |  |  |  |  |  |
| Negative  Positive | 450.0 (86.6) 477.4 (110.7) | 0.103 | 281.3 (60.7)  290.0 (60.1) | 0.458 | 55.6 (18.6)  56.0 (20.6) | 0.912 |

Abbreviations: SD: standard deviation; BMI: body mass index; RT/CRT: radiotherapy/chemoradiotherapy; MMR: mismatch repair.

**Table S6.** Associations of serum leucine, valine and tyrosine levels with clinicopathological characteristics

|  | **Leucine** | | **Valine** | | | | **Tyrosine** | | |
| --- | --- | --- | --- | --- | --- | --- | --- | --- | --- |
|  | **µmol/L, mean (SD)** | **p value** | **µmol/L, mean (SD)** | | **p value** | | **µmol/L, mean (SD)** | | **p value** |
| **Gender** |  |  |  | |  | |  | |  |
| Male  Female | 83.2 (25.6)  71.6 (21.2) | **9.0E-6** | 182.9 (50.0)  162.1 (45.4) | | **9.3E-5** | | 58.5 (16.7)  56.2 (16.5) | | 0.218 |
| **Age** |  |  |  | |  | |  | |  |
| < 65 years  ≥ 65 years | 79.9 (25.0)  76.6 (23.8) | 0.233 | 170.3 (48.8)  174.9 (49.1) | | 0.413 | | 54.8 (15.6)  58.9 (17.1) | | 0.031 |
| **BMI (kg/m^2^)** |  |  |  | |  | |  | |  |
| <20  20-25  25-30  30-35  ≥35 | 62.0 (22.6)  78.1 (25.1)  79.5 (24.2)  79.6 (23.4)  76.5 (23.4) | 0.142 | 144.9 (45.7)  172.6 (48.6)  176.4 (48.5)  181.0 (53.1)  169.0 (46.1) | | 0.160 | | 52.1 (20.3)  57.4 (17.7)  57.5 (15.3)  61.3 (18.6)  55.8 (12.2) | | 0.366 |
| **Tumor location** |  |  |  | |  | |  | |  |
| Proximal  Distal  Rectum | 74.5 (22.4)  78.9 (24.8)  80.1 (24.8) | 0.155 | 167.4 (47.2)  172.5 (52.2)  180.9 (47.0) | | 0.095 | | 57.5 (16.1)  58.5 (15.9)  58.0 (16.9) | | 0.945 |
| **Preoperative RT/CRT in rectal cancers** | | |  | |  | |  | |  |
| No  Yes | 76.7 (22.9)  84.3 (26.9) | 0.068 | 171.4 (44.5)  189.4 (51.0) | | 0.024 | | 57.2 (17.9)  58.3 (16.0) | | 0.698 |
| **TNM Stage** |  |  |  | |  | |  | |  |
| Stage I  Stage II  Stage III  Stage IV | 79.0 (21.6)  78.1 (25.6)  79.7 (24.3)  72.2 (22.3) | 0.428 | 175.5 (39.0)  175.7 (55.1)  179.2 (45.4)  160.4 (48.5) | | 0.088 | | 59.3 (16.2)  56.3 (16.4)  60.9 (16.6)  52.4 (14.8) | | 0.025 |
| **Depth of invasion** |  |  | |  | |  | |  | |
| T1  T2  T3  T4 | 81.4 (25.1)  77.9 (21.2)  78.3 (24.6)  74.9 (27.1) | 0.591 | 180.4 (45.1)  176.6 (40.5)  175.7 (50.8)  159.1 (47.0) | | 0.283 | | 67.4 (22.4)  57.8 (14.7)  57.3 (16.0)  58.9 (20.4) | | 0.150 |
| **Nodal metastasis** |  |  | |  | |  | |  | |
| N0  N1  N2 | 78.1 (24.2)  78.2 (23.5)  77.8 (24.2) | 0.972 | 174.8 (49.3)  178.7 (45.8)  169.3 (47.5) | | 0.679 | | 57.2 (16.5)  58.6 (17.7)  59.7 (13.9) | | 0.649 |
| **Distant metastasis** |  |  | |  | |  | |  | |
| M0  M1 | 78.9 (24.0)  72.2 (22.3) | 0.161 | 177.0 (47.7)  160.4 (48.5) | | 0.024 | | 58.8 (16.5)  52.4 (14.8) | | 0.032 |
| **Lymphatic invasion** |  |  |  | |  | |  | |  |
| No  Yes | 79.4 (24.7)  76.1 (23.1) | 0.205 | 174.9 (48.7)  171.8 (47.5) | | 0.565 | | 57.9 (17.6)  57.1 (15.4) | | 0.680 |
| **Blood vessel invasion** |  |  |  | |  | |  | |  |
| No  Yes | 78.4 (24.3)  75.3 (22.3) | 0.390 | 175.5 (48.6)  163.4 (44.7) | | 0.091 | | 57.5 (16.6)  57.5 (16.4) | | 0.995 |
| **WHO Grade 1-3** |  |  |  | |  | |  | |  |
| Grade 1  Grade 2  Grade 3 | 79.8 (28.0)  78.1 (22.9)  73.7 (21.4) | 0.292 | 174.9 (49.4)  175.1 (47.6)  173.2 (50.0) | | 0.922 | | 58.5 (17.4)  57.6 (16.6)  58.9 (13.8) | | 0.832 |
| **MMR screening status** |  |  | |  | |  | |  | |
| MMR deficient  MMR proficient | 74.1 (24.3)  78.4 (24.2) | 0.305 | 165.7 (55.6)  174.5 (47.8) | | 0.293 | | 56.2 (15.7)  57.7 (16.7) | | 0.597 |
| **BRAF VE1 immunohistochemistry** | |  |  | |  | |  | |  |
| Negative  Positive | 78.1 (24.2)  75.6 (25.8) | 0.587 | 173.8 (48.9)  168.5 (50.7) | | 0.571 | | 57.3 (16.7)  29.7 (16.3) | | 0.443 |

Abbreviations: SD: standard deviation; BMI: body mass index; RT/CRT: radiotherapy/chemoradiotherapy; MMR: mismatch repair.

**Table S7.** Correlation between serum amino acid levels and tumor infiltrating inflammatory cells

|  |  |  | **CD3 IM** | **CD3 CT** | **CD3 IEL** | **CD8 IM** | **CD8 CT** | **CD 8 IEL** | **FoxP3 IM** | **FoxP3**  **CT** | **Mast**  **IM** | **Mast**  **CT** | **Neut**  **IM** | **Neut**  **CT** |
| --- | --- | --- | --- | --- | --- | --- | --- | --- | --- | --- | --- | --- | --- | --- |
| **Ala** | Unadjusted | Pearson r | 0.060 | 0.088 | 0.107 | 0.036 | -0.026 | 0.048 | 0.066 | 0.079 | 0.006 | 0.009 | 0.036 | 0.056 |
|  |  | p value | 0.276 | 0.108 | 0.053 | 0.517 | 0.636 | 0.388 | 0.229 | 0.148 | 0.920 | 0.871 | 0.518 | 0.314 |
|  | Adjusted | Beta | 0.024 | 0.068 | 0.092 | 0.002 | -0.057 | 0.037 | -0.002 | 0.019 | -0.032 | -0.017 | -0.006 | 0.042 |
|  |  | p value | 0.690 | 0.230 | 0.118 | 0.979 | 0.326 | 0.535 | 0.973 | 0.757 | 0.578 | 0.768 | 0.919 | 0.469 |
| **Gln** | Unadjusted | Pearson r | 0.082 | 0.025 | 0.042 | 0.113 | 0.152 | 0.052 | 0.121 | 0.102 | 0.087 | -0.032 | 0.074 | 0.043 |
|  |  | p value | 0.138 | 0.651 | 0.449 | 0.039 | **0.006** | 0.356 | 0.027 | 0.065 | 0.113 | 0.563 | 0.182 | 0.444 |
|  | Adjusted | Beta | 0.021 | -0.013 | 0.005 | 0.061 | 0.123 | 0.020 | 0.015 | 0.012 | 0.043 | -0.047 | 0.010 | 0.014 |
|  |  | p value | 0.723 | 0.810 | 0.926 | 0.310 | 0.031 | 0.734 | 0.805 | 0.845 | 0.437 | 0.387 | 0.863 | 0.804 |
| **Gly** | Unadjusted | Pearson r | -0.035 | -0.024 | -0.041 | 0.034 | 0.035 | -0.039 | -0.13 | -0.099 | -0.074 | -0.031 | -0.026 | 0.023 |
|  |  | p value | 0.525 | 0.673 | 0.460 | 0.545 | 0.526 | 0.491 | 0.020 | 0.074 | 0.182 | 0.577 | 0.646 | 0.678 |
|  | Adjusted | Beta | 0.043 | 0.046 | 0.044 | 0.123 | 0.104 | 0.035 | -0.042 | -0.003 | -0.020 | -0.012 | 0.029 | 0.060 |
|  |  | p value | 0.452 | 0.401 | 0.444 | 0.037 | 0.062 | 0.543 | 0.512 | 0.956 | 0.722 | 0.831 | 0.615 | 0.296 |
| **His** | Unadjusted | Pearson r | 0.022 | 0.004 | 0.061 | 0.024 | 0.061 | 0.076 | 0.045 | 0.109 | 0.014 | 0.105 | -0.030 | -0.024 |
|  |  | p value | 0.687 | 0.942 | 0.267 | 0.665 | 0.263 | 0.174 | 0.417 | 0.047 | 0.796 | 0.055 | 0.586 | 0.664 |
|  | Adjusted | Beta | -0.015 | -0.014 | 0.060 | 0.020 | 0.073 | 0.093 | -0.004 | 0.057 | -0.032 | 0.043 | -0.065 | -0.021 |
|  |  | p value | 0.797 | 0.801 | 0.285 | 0.730 | 0.187 | 0.105 | 0.952 | 0.323 | 0.551 | 0.428 | 0.246 | 0.702 |
| **Ile** | Unadjusted | Pearson r | 0.020 | 0.030 | 0.018 | 0.036 | -0.004 | 0.030 | 0.006 | -0.002 | 0.010 | 0.011 | -0.048 | -0.002 |
|  |  | p value | 0.712 | 0.584 | 0.739 | 0.518 | 0.941 | 0.594 | 0.906 | 0.965 | 0.853 | 0.837 | 0.391 | 0.969 |
|  | Adjusted | Beta | 0.010 | 0.024 | 0.011 | 0.033 | 0.004 | 0.035 | -0.016 | -0.034 | -0.005 | -0.003 | -0.064 | -0.008 |
|  |  | p value | 0.863 | 0.668 | 0.852 | 0.586 | 0.942 | 0.552 | 0.802 | 0.578 | 0.936 | 0.953 | 0.273 | 0.894 |
| **Leu** | Unadjusted | Pearson r | -0.002 | 0.053 | 0.033 | -0.006 | -0.022 | 0.005 | -0.009 | 0.023 | -0.018 | 0.050 | -0.041 | -0.019 |
|  |  | p value | 0.968 | 0.331 | 0.547 | 0.912 | 0.693 | 0.928 | 0.864 | 0.682 | 0.749 | 0.364 | 0.464 | 0.735 |
|  | Adjusted | Beta | -0.001 | 0.054 | 0.044 | 0.019 | -0.002 | 0.023 | -0.007 | 0.005 | -0.037 | 0.011 | -0.038 | -0.001 |
|  |  | p value | 0.985 | 0.335 | 0.450 | 0.751 | 0.978 | 0.697 | 0.912 | 0.936 | 0.514 | 0.839 | 0.513 | 0.989 |
| **Val** | Unadjusted | Pearson r | -0.005 | 0.037 | 0.014 | -0.018 | -0.049 | -0.012 | 0.025 | 0.021 | 0.039 | 0.063 | -0.029 | -0.021 |
|  |  | p value | 0.927 | 0.500 | 0.796 | 0.740 | 0.369 | 0.825 | 0.648 | 0.698 | 0.476 | 0.254 | 0.607 | 0.706 |
|  | Adjusted | Beta | 0.005 | 0.034 | 0.026 | 0.018 | -0.034 | 0.010 | 0.025 | -0.011 | 0.020 | 0.025 | -0.029 | 0.004 |
|  |  | p value | 0.933 | 0.541 | 0.655 | 0.762 | 0.549 | 0.869 | 0.686 | 0.857 | 0.721 | 0.656 | 0.611 | 0.940 |
| **Phe** | Unadjusted | Pearson r | -0.016 | -0.005 | -0.006 | 0.050 | -0.045 | -0.037 | -0.116 | -0.077 | -0.054 | -0.029 | -0.006 | 0.057 |
|  |  | p value | 0.777 | 0.924 | 0.915 | 0.365 | 0.417 | 0.505 | 0.034 | 0.160 | 0.324 | 0.599 | 0.912 | 0.304 |
|  | Adjusted | Beta | 0.045 | 0.038 | 0.051 | 0.107 | -0.004 | 0.017 | -0.068 | -0.014 | -0.005 | 0.003 | 0.030 | 0.077 |
|  |  | p value | 0.441 | 0.498 | 0.376 | 0.073 | 0.939 | 0.766 | 0.274 | 0.812 | 0.927 | 0.958 | 0.603 | 0.176 |
| **Tyr** | Unadjusted | Pearson r | -0.006 | -0.027 | -0.039 | 0.037 | 0.010 | 0.027 | 0.014 | 0.034 | 0.055 | 0.025 | 0.025 | 0.042 |
|  |  | p value | 0.911 | 0.626 | 0.475 | 0.501 | 0.849 | 0.631 | 0.796 | 0.541 | 0.319 | 0.654 | 0.653 | 0.452 |
|  | Adjusted | Beta | -0.011 | -0.037 | -0.048 | 0.045 | 0.007 | 0.034 | -0.035 | -0.007 | 0.047 | 0.028 | 0.006 | 0.051 |
|  |  | p value | 0.851 | 0.508 | 0.407 | 0.453 | 0.907 | 0.567 | 0.577 | 0.905 | 0.399 | 0.615 | 0.916 | 0.372 |

The correlations were adjusted for age, tumor location (colon vs. rectum), tumor stage variables (T1-2 vs. T3-4; N0 vs. N1-2; M0 vs. M1) and preoperative radiotherapy/chemoradiotherapy (no vs. yes). Abbreviations: IM: invasive margin; CT-S, center of the tumor, stroma; CT-IEL, center of the tumor, intraepithelial; Ala: alanine; Gln: glutamine; Gly: glycine; His: histidine; Ile: isoleucine; Leu: leucine, Val: valine; Phe: phenylalanine; Tyr: tyrosine. Inflammatory cell counts were logarithmically transformed.

**Table S8.** Serum amino acid levels in relation to mGPS in stage I-III CRC patients.

|  | mGPS 0 (n=240) | mGPS 1-2 (n=48) |  |  |
| --- | --- | --- | --- | --- |
|  | **µmol/L, mean (SD)** | **µmol/L, mean (SD)** | **p value** | **Adjusted p value** |
| Alanine | 463.7 (91.6) | 434.8 (75.1) | 0.093 | 0.124 |
| Glutamine | 501.8 (78.4) | 469.8 (90.1) | **0.005** | **0.008** |
| Glycine | 277.3 (57.1) | 277.4 (49.8) | 0.783 | 0.745 |
| Histidine | 59.9 (9.4) | 50.8 (10.1) | **9.1E-10** | **2.8E-9** |
| Isoleucine | 57.4 (18.7) | 53.4 (16.7) | 0.346 | 0.258 |
| Leucine | 80.8 (24.0) | 71.5 (22.3) | 0.053 | 0.065 |
| Valine | 181.3 (46.6) | 158.4 (46.1) | **0.002** | 0.012 |
| Phenylalanine | 82.0 (12.5) | 92.7 (25.3) | **1.2E-4** | **3.0E-6** |
| Tyrosine | 59.7 (16.6) | 54.3 (15.2) | 0.102 | 0.153 |

Abbreviations: mGPS: modified Glasqow Prognostic Score; SD: standard deviation. P values were adjusted for tumor location (colon vs. rectum), tumor stage variables (T1-2 vs. T3-4; N0 vs. N1-2) and preoperative radiotherapy/chemoradiotherapy (no vs. yes).

**Table S9.** Serum amino acid levels in relation to mGPS in stage IV CRC patients.

|  | mGPS 0 (n=22) | mGPS 1-2 (n=23) |  |  |
| --- | --- | --- | --- | --- |
|  | **µmol/L, mean (SD)** | **µmol/L, mean (SD)** | **p value** | **Adjusted p value** |
| Alanine | 411.8 (63.6) | 427.9 (95.3) | 0.428 | 0.423 |
| Glutamine | 498.2 (81.1) | 396.0 (94.4) | **0.001** | **0.007** |
| Glycine | 306.0 (94.4) | 314.2 (57.5) | 0.456 | 0.936 |
| Histidine | 55.8 (10.8) | 48.2 (8.6) | **0.005** | 0.028 |
| Isoleucine | 47.4 (14.0) | 52.3 (19.5) | 0.814 | 0.569 |
| Leucine | 65.6 (18.2) | 74.9 (26.1) | 0.308 | 0.151 |
| Valine | 152.5 (44.6) | 162.4 (58.7) | 0.319 | 0.106 |
| Phenylalanine | 75.7 (12.4) | 102.1 (19.9) | **4.1E-4** | **0.002** |
| Tyrosine | 49.5 (14.1) | 54.8 (15.4) | 0.222 | 0.446 |

Abbreviations: mGPS: modified Glasqow Prognostic Score; SD: standard deviation. P values were adjusted for tumor location (colon vs. rectum), tumor stage variables (T1-2 vs. T3-4; N0 vs. N1-2) and preoperative radiotherapy/chemoradiotherapy (no vs. yes).

**Table S10.** Multiple linear regression model of serum amino acid levels in colorectal cancer patients

| **Amino acid** | **Independent** | **Beta** | **p value** |
| --- | --- | --- | --- |
| **Histidine** | **Model 1** |  |  |
|  | mGPS | -0.380 | **7.6E-13** |
|  | **Model 2** |  |  |
|  | mGPS | -0.372 | **1.9E-12** |
|  | Gender | -0.113 | 0.026 |
| **Phenylalanine** | **Model 1** |  |  |
|  | mGPS | 0.365 | **6.4E-12** |
| **Alanine** | **Model 1** |  |  |
|  | Distant metastasis | -0.135 | 0.014 |
| **Glycine** | **Model 1** |  |  |
|  | Gender | 0.273 | **6.0E-7** |
|  | **Model 2** |  |  |
|  | Gender | 0.254 | **3.0E-6** |
|  | Nodal metastasis | 0.172 | **0.001** |
|  | **Model 3** |  |  |
|  | Gender | 0.248 | **5.0E-6** |
|  | Nodal metastasis | 0.136 | 0.015 |
|  | Distant metastasis | 0.116 | 0.037 |
| **Valine** | **Model 1** |  |  |
|  | Gender | -0.200 | **2.4E-4** |
|  | **Model 2** |  |  |
|  | Gender | -0.192 | **4.2E-4** |
|  | Distant metastasis | -0.113 | 0.037 |

Abbreviations: mGPS: modified Glasgow Prognostic Score.

**Table S11.** Receiver operating characteristics (ROC) analysis for determining cut-off points in detecting cancer-specific survival

|  | AUC | 95% CI | Cut-off point (µmol/L) |
| --- | --- | --- | --- |
| Glutamine | 0.562 | 0.482-0.641 | 410.0 |
| Histidine | 0.586 | 0.510-0.661 | 55.0 |
| Phenylalanine | 0.591 | 0.507-0.674 | 93.0 |
| Alanine | 0.529 | 0.452-0.607 | 445.0 |
| Glycine | 0.648 | 0.571-0.725 | 263.0 |
| Isoleucine | 0.528 | 0.453-0.602 | 53.0 |
| Leucine | 0.512 | 0.438-0.587 | 74.0 |
| Valine | 0.530 | 0.453-0.606 | 169.0 |
| Tyrosine | 0.502 | 0.426-0.578 | 57.0 |

Abbreviations: AUC: area under curve; CI: confidence interval

**Table S12.** Univariate analysis of disease-free survival (DFS), cancer-specific survival (CSS) and overall survival (OS) according to serum amino acid levels

|  | DFS | | | CSS | | | OS | | |
| --- | --- | --- | --- | --- | --- | --- | --- | --- | --- |
|  | **HR** | **95% CI** | **p value** | **HR** | **95% CI** | **p value** | **HR** | **95% CI** | **p value** |
| Glutamine (<410 vs. ≥410 µmol/L) | 1.27 | 0.58-2.80 | 0.555 | 0.46 | 0.28-0.78 | **0.004** | 0.53 | 0.35-0.83 | **0.005** |
| Histidine (<55 vs. ≥55 µmol/L) | 1.48 | 0.81-2.71 | 0.200 | 0.47 | 0.29-0.75 | **0.001** | 0.51 | 0.35-0.75 | **0.001** |
| Phenylalanine (<93 vs. ≥93 µmol/L) | 1.58 | 0.89-2.80 | 0.119 | 2.74 | 1.72-4.36 | **2.4E-5** | 2.29 | 1.55-3.38 | **3.4E-5** |
| Alanine (<445 vs. ≥445 µmol/L) | 1.72 | 1.01-2.93 | 0.046 | 0.97 | 0.61-1.55 | 0.911 | 0.77 | 0.53-1.13 | 0.188 |
| Glycine (<263 vs. ≥263 µmol/L) | 1.87 | 1.09-3.23 | 0.024 | 2.39 | 1.41-4.05 | **0.001** | 1.44 | 0.97-2.15 | 0.072 |
| Isoleucine (<53 vs. ≥53 µmol/L) | 0.80 | 0.48-1.34 | 0.395 | 0.60 | 0.37-0.96 | 0.032 | 0.69 | 0.47-1.02 | 0.060 |
| Leucine (<74 vs. ≥74 µmol/L) | 0.93 | 0.56-1.55 | 0.782 | 0.89 | 0.56-1.42 | 0.625 | 0.88 | 0.60-1.29 | 0.497 |
| Valine (<169 vs. ≥169 µmol/L) | 0.91 | 0.62-1.72 | 0.911 | 0.70 | 0.44-1.12 | 0.141 | 0.75 | 0.51-1.11 | 0.149 |
| Tyrosine (<57 vs. ≥57 µmol/L) | 1.34 | 0.80-2.42 | 0.266 | 0.94 | 0.59-1.50 | 0.799 | 0.82 | 0.56-1.21 | 0.321 |

Abbreviations: CI: confidence interval; HR: hazard ratio

**Table S13.** Multivariate analysis of cancer-specific survival (CSS) according to clinicopathological parameters and serum glutamine level

|  |  | CSS |  |
| --- | --- | --- | --- |
|  | **HR** | **95%CI** | **p value** |
| Age (<65 vs. ≥65) | 1.89 | 1.09-3.27 | 0.023 |
| Preoperative RT/CRT (No vs. Yes) | 1.12 | 0.53-2.35 | 0.770 |
| Localisation (Colon vs. Rectum) | 0.89 | 0.51-1.54 | 0.668 |
| Tumor invasion (T1-T2 vs. T3-T4) | 0.80 | 0.41-1.58 | 0.522 |
| Nodal metastases (N0 vs. N1-N2) | 3.65 | 1.80-7.41 | 0.000 |
| Distant metastases (M0 vs. M1) | 8.24 | 4.49-15.12 | 0.000 |
| Lymphatic invasion (No vs. Yes) | 1.98 | 1.00-3.93 | 0.051 |
| Grade (1-2 vs. 3) | 1.90 | 1.21-2.97 | 0.005 |
| mGPS (0 vs. 1-2) | 1.11 | 0.59-2.08 | 0.753 |
| Glutamine (<410 vs. ≥410 µmol/L) | 0.63 | 0.32-1.21 | 0.164 |

CSS: n=324; median follow-up time 65.7 months (IQR 36.7-85.6); 68 (20.2%) events; 10 (3.0%) cases excluded from the analysis because of missing values. Abbreviations: CI: confidence interval; HR: hazard ratio; RT/CRT: radiotherapy or chemoradiotherapy.

**Table S14.** Multivariate analysis of cancer-specific survival (CSS) according to clinicopathological parameters and serum histidine level

|  |  | CSS |  |
| --- | --- | --- | --- |
|  | **HR** | **95%CI** | **p value** |
| Age (<65 vs. ≥65) | 1.86 | 1.07-3.21 | 0.027 |
| Preoperative RT/CRT (No vs. Yes) | 1.26 | 0.59-2.69 | 0.551 |
| Localisation (Colon vs. Rectum) | 0.87 | 0.49-1.53 | 0.628 |
| Tumor invasion (T1-T2 vs. T3-T4) | 0.87 | 0.44-1.70 | 0.681 |
| Nodal metastases (N0 vs. N1-N2) | 3.42 | 1.68-6.97 | 0.001 |
| Distant metastases (M0 vs. M1) | 7.52 | 4.03-14.03 | 0.000 |
| Lymphatic invasion (No vs. Yes) | 1.96 | 0.98-3.92 | 0.057 |
| Grade (1-2 vs. 3) | 1.75 | 1.18-2.59 | 0.006 |
| mGPS (0 vs. 1-2) | 1.16 | 0.63-2.12 | 0.632 |
| Histidine (<55 vs. ≥ 55 µmol/L) | 0.85 | 0.48-1.50 | 0.567 |

CSS: n=323; median follow-up time 65.7 months (IQR 36.7-85.6); 69 (20.5%) events; 11 (3.3%) cases excluded from the analysis because of missing values. Abbreviations: CI: confidence interval; HR: hazard ratio; RT/CRT: radiotherapy or chemoradiotherapy.

**Table S15.** Multivariate analysis of cancer-specific survival (CSS) according to clinicopathological parameters and serum phenylalanine level

|  |  | CSS |  |
| --- | --- | --- | --- |
|  | **HR** | **95%CI** | **p value** |
| Age (<65 vs. ≥65) | 1.76 | 1.02-3.05 | 0.042 |
| Preoperative RT/CRT (No vs. Yes) | 1.22 | 0.57-2.59 | 0.608 |
| Localisation (Colon vs. Rectum) | 0.91 | 0.52-1.58 | 0.728 |
| Tumor invasion (T1-T2 vs. T3-T4) | 0.86 | 0.44-1.68 | 0.663 |
| Nodal metastases (N0 vs. N1-N2) | 3.21 | 1.59-6.46 | 0.001 |
| Distant metastases (M0 vs. M1) | 7.87 | 4.27-14.53 | 0.000 |
| Lymphatic invasion (No vs. Yes) | 2.00 | 1.01-3.98 | 0.048 |
| Grade (1-2 vs. 3) | 1.75 | 1.18-2.60 | 0.005 |
| mGPS (0 vs. 1-2) | 1.03 | 0.54-1.98 | 0.933 |
| Phenylalanine (<93 vs. ≥93 µmol/l) | 1.54 | 0.86-2.75 | 0.144 |

CSS: n=327; median follow-up time 65.7 months (IQR 36.7-85.6); 70 (20.8%) events; 7 (2.1%) cases excluded from the analysis because of missing values. Abbreviations: CI: confidence interval; HR: hazard ratio; RT/CRT: radiotherapy or chemoradiotherapy.

**Table S16.** Multivariate analysis of cancer-specific survival (CSS) according to clinicopathological parameters and serum glycine level

|  |  | CSS |  |
| --- | --- | --- | --- |
|  | **HR** | **95%CI** | **p value** |
| Age (<65 vs. ≥65) | 1.91 | 1.11-3.30 | 0.020 |
| Preoperative RT/CRT (No vs. Yes) | 1.10 | 0.51-2.38 | 0.811 |
| Localisation (Colon vs. Rectum) | 0.98 | 0.55-1.76 | 0.951 |
| Tumor invasion (T1-T2 vs. T3-T4) | 1.05 | 0.51-2.13 | 0.901 |
| Nodal metastases (N0 vs. N1-N2) | 2.85 | 1.40-5.81 | 0.004 |
| Distant metastases (M0 vs. M1) | 7.07 | 3.83-13.06 | 0.000 |
| Lymphatic invasion (No vs. Yes) | 2.42 | 1.17-5.01 | 0.018 |
| Grade (1-2 vs. 3) | 1.86 | 1.24-2.80 | 0.003 |
| mGPS (0 vs. 1-2) | 1.19 | 0.66-2.17 | 0.566 |
| Glycine (<263 vs. ≥263 µmol/L) | 1.36 | 0.77-2.40 | 0.289 |

CSS: n=317; median follow-up time 65.7 months (IQR 36.7-85.6); 68 (20.2%) events; 17 (5.1%) cases excluded from the analysis because of missing values. Abbreviations: CI: confidence interval; HR: hazard ratio; RT/CRT: radiotherapy or chemoradiotherapy.
