# Supplementary material for: Alterations in serum amino-acid profile in the progression of colorectal cancer: associations with systemic inflammation, tumour stage and patient survival
Source: Br J Cancer. 2018 Dec 19;120(2):238–46. doi: 10.1038/s41416-018-0357-6 (PMC6342921; doi:10.1038/s41416-018-0357-6)
Supplement: Supplementary file 2 — Supplementary Figure S1 [file 41416_2018_357_MOESM2_ESM.docx]

**Figure S1.** Correlation matrix plot displaying interrelationships between different serum amino acids. Positive correlations are shown in blue and negative correlations in red. Color intensity and the size of the circle are proportional to the Pearson correlation coefficients. *** = p < 0.001, ** = p < 0.01, * = p < 0.05.
